# The Book World of Medicine and Science

**Published:** 1903-09-26

**Authors:** 


### The Book World of Medicine and Science.

The Health Resorts of Europe. A Medical Guide to the Mineral Springs, Climatic Mountain and Seaside Health Resorts, Milk, Whey, Grape, Earth, Mud, Sand and Air Cures of Europe. By Thomas Linn, M.D. (Eleventh edition. Pp. 258. London: The Health Resorts Bureau. 1903. Price 2s. 6d. net.)

Dr. Linn's convenient little guide to the more important health stations of Europe is so well known as to call for but scant notice. In the present edition the general character of the volume remains unchanged although considerable pains have evidently been taken to bring it up-to-date. Much common-sense advice is afforded respecting the selection of a suitable health resort and particulars are given of the various kinds of so-called "cures." The greater portion of the volume is taken up with a description of the various health resorts according to their countries, and the alphabetical arrangement adopted makes it very handy for ready reference. Every page is rich in serviceable particulars and they seem generally to be well selected and reliable. We think it a pity that little more than the names of sanatoria for consumption is mentioned; descriptions of the chief features of the more important establishments for the open-air treatment of pulmonary tuberculosis might well have been afforded. In appendices particulars are supplied respecting the British Balneological and Climatological Society, the Continental Anglo-American Medical Society, the American Dental Society of Europe, and lists of hydropathic establishments, sanatoria and private cliniques are also presented. It is a serious drawback that no good map is included in the volume. Although guilty of many omissions, the volume is one which may well be kept within ready reach for purposes of convenient consultation respecting the more frequented European health resorts and we commend it to the notice of those not already acquainted with its many excellencies.

A Skin Pharmacopœia. By J. Startin, Senior Surgeon to the London Skin Hospital. 5th Edition. (Bristol: Wright. 1903. Pp. 64. 2s. 6d.)

The many affections of the skin met with both in hospital and general practice are often as puzzling as is the dermatologist's nomenclature itself. It is certain that neither from plates nor from descriptions can be acquired the principles of reliable diagnosis; hence the desirability of studying these affections in the skin departments of our hospitals, or in hospitals set apart wholly for the treatment of disease of the skin. And as the sufferer is particularly conscious of his complaint, the progress of which he can himself see, this branch of medicine is one which should be especially appreciated by students, owing to its usefulness in practice. The appearance of the fifth edition of Mr. Startin's little pharmacopœia may be taken as a warranty of its value; but, however excellent it may be, its usefulness, unaided by clinical study, would be considerably lessened. It contains concise formulæ for baths, mixtures, ointments, lotions, etc., and rules of diet, with a therapeutic index, all of which are good; and though we think a simpler and more uniform system of proportions in formulæ of prescriptions might be an advantage, we scarcely think the sphere of usefulness of the book is likely to be materially diminished by the system adopted.

Polyphase Currents in Electro-therapy. By George Herschell, M.D.Lond. (London: Glaisher. 1903. Pp. 44. Numerous illustrations.)

This book represents a contribution to our knowledge of electro-therapeutics. In medicine but little attention has been paid to the polyphase system, especially in this country, though polyphase electric currents are employed industrially on account of their economy. Those familiar with the production of an alternating single phase have no difficulty in comprehending the nature of the polyphase currents. The writer, quoting Oudin, defines the system thus: "Any arrangement of conductors carrying two or more currents definitely related to one another in point of time constitutes a polyphase system." The triphase system appears to be the one most suitable for therapeutic work, owing to the property of the currents to produce a rotating magnetic field. The tissues under their influence become included in *un tourbillon électrique*, as Gumbail terms it. The author illustrates the physiological effects of triphase currents by showing pulse-tracings in six cases, before and after application. In each, increase in tension and amplitude are demonstrated; but among the most remarkable properties claimed as distinctive of these currents is their action on unstriped muscle fibre of the alimentary canal, and there appears to be no doubt that the stomach and intestines are awakened into renewed activity under their influence.

Radium and other Radio-active Substances. By William J. Hammer. (London: Sampson Low, Marston and Company, Limited. Pp. 72. Price 5s. net.)

This book, or rather pamphlet, is divided into five chapters, which deal respectively with fluorescence, phosphorescence, radium, polonium, actinium and thorium, the properties and applications of selenium, and the treatment of disease by ultra-violet rays. It must be confessed that the author's method of dealing with these interesting subjects is inclined to be somewhat confusing and difficult to follow, and moreover the reader cannot quite free himself from the desire to have a few more facts in exchange for some of the very numerous names that are introduced. Probably most medical readers will agree that the last chapter might have been omitted without seriously affecting the value of the work. Doubless there are points in the pamphlet which will be of value to electricians and others who are specially concerned with the subjects to which the work refers.

How to Take Care of a Consumptive. By M. Forrest Williams. (London: John Long. 1903. Pp. 47. Price 1s. net.)

This unpretentious but well-meaning booklet, written manifestly by a non-medical enthusiast, seeks to awaken interest in a rational application of hygienic measures to the treatment of pulmonary tuberculosis. It deals with many of the points which are all essential in the so-called "open-air" method in a manner interesting and attractive, although somewhat desultory and lacking in scientific precision. It requires much experience and exceptional judgment to write a book on such a subject as this which may present necessary truth concerning a serious subject in simple terms and yet without possibility of misunderstanding; and although this little handbook will doubtless be of service it must be read with a discriminating care.

